# Genome-Wide Analysis of the MYB-Related Transcription Factor Family in Pepper and Functional Studies of *CaMYB37* Involvement in Capsaicin Biosynthesis

**DOI:** 10.3390/ijms231911667

**Published:** 2022-10-01

**Authors:** Yi Liu, Zhishuo Zhang, Ke Fang, Qingyun Shan, Lun He, Xiongze Dai, Xuexiao Zou, Feng Liu

**Affiliations:** 1 Longping Branch, Graduate School of Hunan University, Changsha 410125, China; 2 Key Laboratory for Vegetable Biology of Hunan Province, Engineering Research Center for Germplasm Innovation and New Varieties Breeding of Horticultural Crops, College of Horticulture, Hunan Agricultural University, Changsha 410125, China

**Keywords:** genome-wide, pepper, *CaMYB*-related transcription factor family, phylogenetic relationships, capsaicin biosynthesis

## Abstract

Chili pepper is an important economic vegetable worldwide. *MYB* family gene members play an important role in the metabolic processes in plant growth and development. In this study, 103 pepper *MYB*-related members were identified and grouped into nine subfamilies according to phylogenetic relationships. Additionally, a total of 80, 20, and 37 collinear gene pairs were identified between pepper and tomato, pepper and *Arabidopsis*, and tomato and *Arabidopsis*, respectively. We performed promoter *cis*-element analysis and showed that *CaMYB*-related members may be involved in multiple biological processes such as growth and development, secondary metabolism, and circadian rhythm regulation. Expression pattern analysis indicated that *CaMYB37* is significantly more enriched in fruit placenta, suggesting that this gene may be involved in capsaicin biosynthesis. Through VIGS, we confirmed that *CaMYB37* is critical for the biosynthesis of capsaicin in placenta. Our subcellular localization studies revealed that CaMYB37 localized in the nucleus. On the basis of yeast one-hybrid and dual-luciferase reporter assays, we found that CaMYB37 directly binds to the promoter of capsaicin biosynthesis gene *AT3* and activates its transcription, thereby regulating capsaicin biosynthesis. In summary, we systematically identified members of the *CaMYB*-related family, predicted their possible biological functions, and revealed that *CaMYB37* is critical for the transcriptional regulation of capsaicin biosynthesis. This work provides a foundation for further studies of the CaMYB-related family in pepper growth and development.

## 1. Introduction

Transcription factors play an important role in regulating plant growth and development [1]. *MYB* transcription factors are ubiquitous in eukaryotes and comprise one of the largest transcription factor families [2,3]. Members of the *MYB* family are involved in the regulation of plant growth, development, and metabolism. For example, overexpression of *EgMYB2* in tobacco leads to increased lignin content and secondary cell-wall thickening [4]. *MYB* transcription factors in tea plants may regulate theanine synthesis in different organs and tissues [5]. *Arabidopsis MYB12* closely regulates the content of flavonols [6]. In apple, *MdMYB1* interacts with transcription factor *MdGSTF6* to regulate anthocyanin synthesis and transport [7]. In apple, *MdMYB6* also inhibits the biosynthesis of anthocyanins by reducing the contents of UDP-glucose and UDP-galactose [8]. In R2R3-*MYB*-type transcriptional activator *WP1*, the C-terminal acidic activation motif can directly regulate the expression of carotenoid biosynthesis genes, such as *MtLYCb* [9]. *MYB* transcription factors usually interact with transcription factors such as *WD40* and *bHLH* to regulate the biosynthesis of anthocyanins. PacMYBA binds to the promoters of *DFR*, *ANS*, and *UFGT* after interacting with several *bHLH* family members in sweet cherry [10]. Silencing *BvMYB1* in sugar beet can downregulate the expression of genes related to betaine biosynthesis and reduce pigmentation [11].

A typical MYB transcription factor contains one or more MYB domains. The MYB domain is characterized by three helices [12], in which the two C-terminal helices form a helix–turn–helix structure (HTH) [13]. The HTH structure recognizes the target motif C/TAACG/TG and provides specificity of MYB [14]. The N-terminal first helix contains a DNA-binding region and is usually composed of several tandem nonrepetitive R structures, with each R structure containing about 51 amino acids. The transcriptional regulatory region at the C-terminus is highly diverse. This may explain the diversified functions of *MYB* transcription factors [15]. According to the number of tandem structures of DNA-binding domains, the MYB transcription factor family can be divided into four subfamilies: 1R-MYB (MYB-Related), R2R3-MYB, 3R-MYB, and 4R-MYB. The R2R3 subfamily has the largest number of members, followed by the 1R subfamily [3].

*Capsicum*, a crop that originates from tropical regions of Latin America, is an important member of the nightshade family along with tomato, tobacco, potato, and petunia [16]. Pepper has important economic value and is grown worldwide [17]. Pepper has five domesticated species, which are highly different in their spiciness [18]. The spiciness mainly comes from capsaicinoids, economically important alkaloids [19,20]. Capsaicinoids are specifically synthesized in the glandular cells of the fruit placenta and then transported to other organs [21]. About 24 species of capsaicinoids have been identified, of which capsaicin (Cap) and dihydrocapsaicin (DhCap) account for over 90% of the total content and are the main determinants of spiciness [22]. The spicy taste produced by eating chili is essentially the burning sensation caused by the binding of capsaicin to the capsaicin receptor TRPV1 in the human neurosensory system [23]. Purified capsaicinoids have an important pharmaceutical, military, and food processing role [24]. Capsaicinoids can be synthesized through two intermate pathways. Phenylalanine is catalyzed by a series of enzymes to derive vanillylamine, and the key enzymes involved in this pathway are phenylalanine ammonialyase (PAL), cinnamic acid hydrolase (C4H), *p*-coumaric acid hydrolase (C3H), caffeic acid transmethoxylase (CoMT), aminotransferase (AMT), β-ketoacyl-ACP synthase (Kas), etc. Valine is catalyzed by a series of enzymes to derive branched-chain fatty acids of 8-methyl-6-nonene-CoA. The key enzymes involved in this pathway are branched-chain amino-acid transferase (BCAT) and isovalerate dehydrogenase (IVD), acyl transportin (ACL), acyl-ACP-thioesterase (FatA), and acyl-CoA synthase (ACS). 8-methyl-6-nonenoyl-CoA and vanillylamine form capsaicin catalyzed by capsaicin synthase (CS) [21,22,25].

Previous studies showed that *R2R3-MYB* genes are involved in the regulation of biocapsaicin synthesis. For instance, *MYB31* upregulates *CBGs* functions to increase capsaicin accumulation by promoting the expression level [18]. Silencing *MYB48* reduced the expression of key capsaicin biosynthetic enzymes, including *AT3* and *KAS*, and affected the biosynthesis of capsaicin [26]. However, little is known about how *CaMYB*-related genes regulate capsaicin biosynthesis.

In this study, we identified 103 *CaMYB*-related members in the pepper genome and revealed their chromosomal locations, phylogenetic relationships, promoter *cis*-elements, and expression patterns. Transcriptome analysis showed that *CaMYB37* was specifically expressed in the fruit placenta and flower. Silencing of *CaMYB37* resulted in a significant decrease in the capsaicin content. Y1H and dual-luciferase reporter assays proved that CaMYB37 can directly bind to the *AT3* promoter and activate its transcription, thereby promoting capsaicin biosynthesis. This study provides a foundation for further research on the functional mechanism of *CaMYB*-related family members involved in pepper growth, development, and metabolism.

## 2. Results

### 2.1. Identification and Protein Characterization of the CaMYB-Related Family

A total of 103 *CaMYB*-related genes and 132 *SlMYB*-related genes were obtained for further analysis (Supplementary Table S1). CaMYB-related proteins range from 101 amino acids (Capana04g000348) to 1260 amino acids (Capana09g002226), with the isoelectric point PI ranging from 4.53 (Capana05g000377) to 11.39 (Capana05g001626), and the MW molecular weight ranging from 11.3 kDa (Capana04g000348) to 140.9 kDa (Capana09g002226) (Supplementary Table S2).

### 2.2. Chromosomal Localization of the CaMYB-Related Family

**Figure 1:**
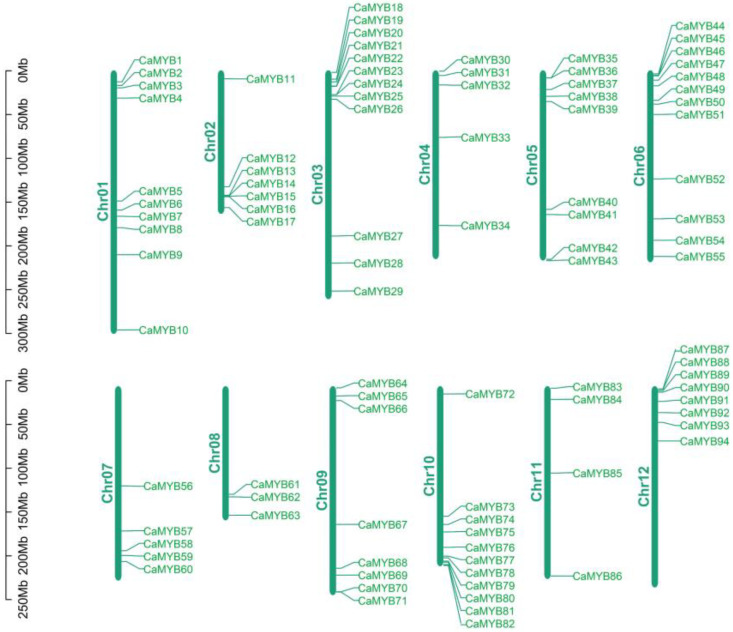
Chromosomal locations of *CaMYB*-related genes.

All 103 *CaMYB*-related transcription factors are named according to their chromosomal location (*CaMYB1*–*CaMYB103*) (Figure 1). According to published genome sequences, 94 *CaMYB*-related genes are distributed on 12 chromosomes (91%), whereas 9 *CaMYB*-related genes stayed on as yet unmapped scaffolds. Most *CaMYB*-related genes are clustered at the two ends of the chromosome, while fewer genes are distributed in the middle of the chromosome.

### 2.3. Duplication Analysis of Members of the CaMYB-Related Family

**Figure 2:**
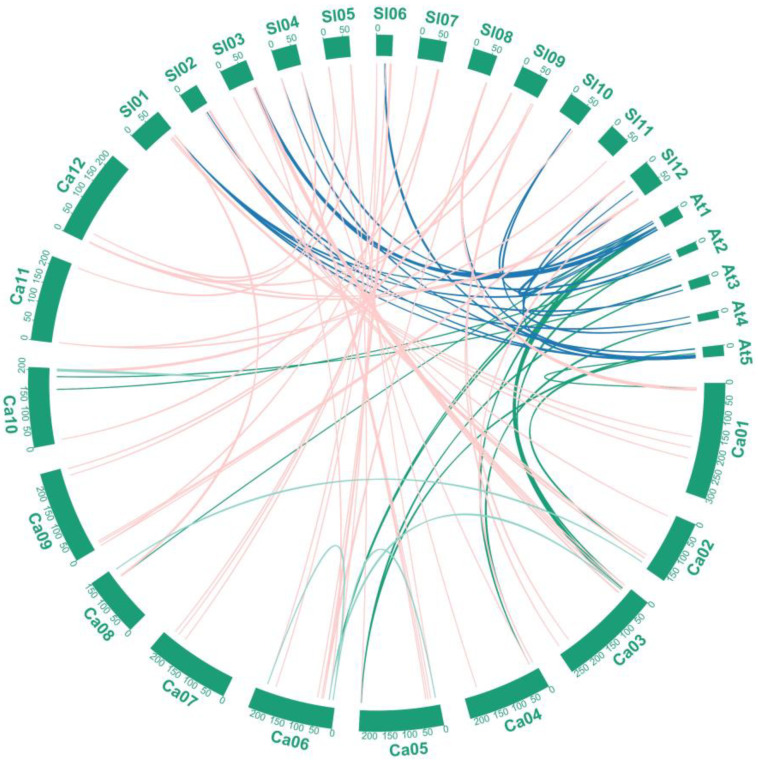
Collinear analysis of *CaMYB*-related genes among pepper (Ca), *Arabidopsis* (At), and tomato (Sl). Blue, green, pink, and blue-green lines represent the collinear gene pairs between chromosomes of tomato and *Arabidopsis*, *Arabidopsis* and pepper, tomato and pepper, and pepper and pepper, respectively. Chromosome numbers are located above the chromosomes.

To further explore the evolutionary relationship between *MYB*-related genes in different species, we analyzed the gene pairs within pepper and those with tomato and *Arabidopsis* (Figure 2). The intraspecific collinearity of pepper showed that six gene pairs may have undergone segmental duplication in evolution: *CaMYB48/CaMYB21*, *CaMYB46/CaMYB35*, *CaMYB54/CaMYB46, CaMYB82/CaMYB79*, *CaMYB63/CaMYB17*, and *CaMYB81/CaMYB80*. The number of *MYB*-related gene pairs between pepper and tomato (80) was higher than that between pepper and *Arabidopsis* (20) and between tomato and *Arabidopsis* (37). This finding indicates that tomato and pepper are more closely related. We found that 64 *CaMYB*-related genes were collinear with 63 *SlMYB*-related genes, most of which were one-to-one, such as *CaMYB44/SlMYB71*; *CaMYB34/SlMYB47*. We also found one-to-many cases: *CaMYB21/(SlMYB29, SlMYB67)* and *CaMYB89/(SlMYB92, SlMYB131)*. Many-to-one cases also existed such as *(CaMYB63, CaMYB17)/SlMYB11* and *(CaMYB79, CaMYB82)/SlMYB101*. These results indicate that MYB-related genes may have evolved from the same ancestor; accordingly, they possess similar functions and, thus, are evolutionarily conserved (Supplementary Table S3).

### 2.4. Structure and Motif Analysis of CaMYB-Related Family

**Figure 3:**
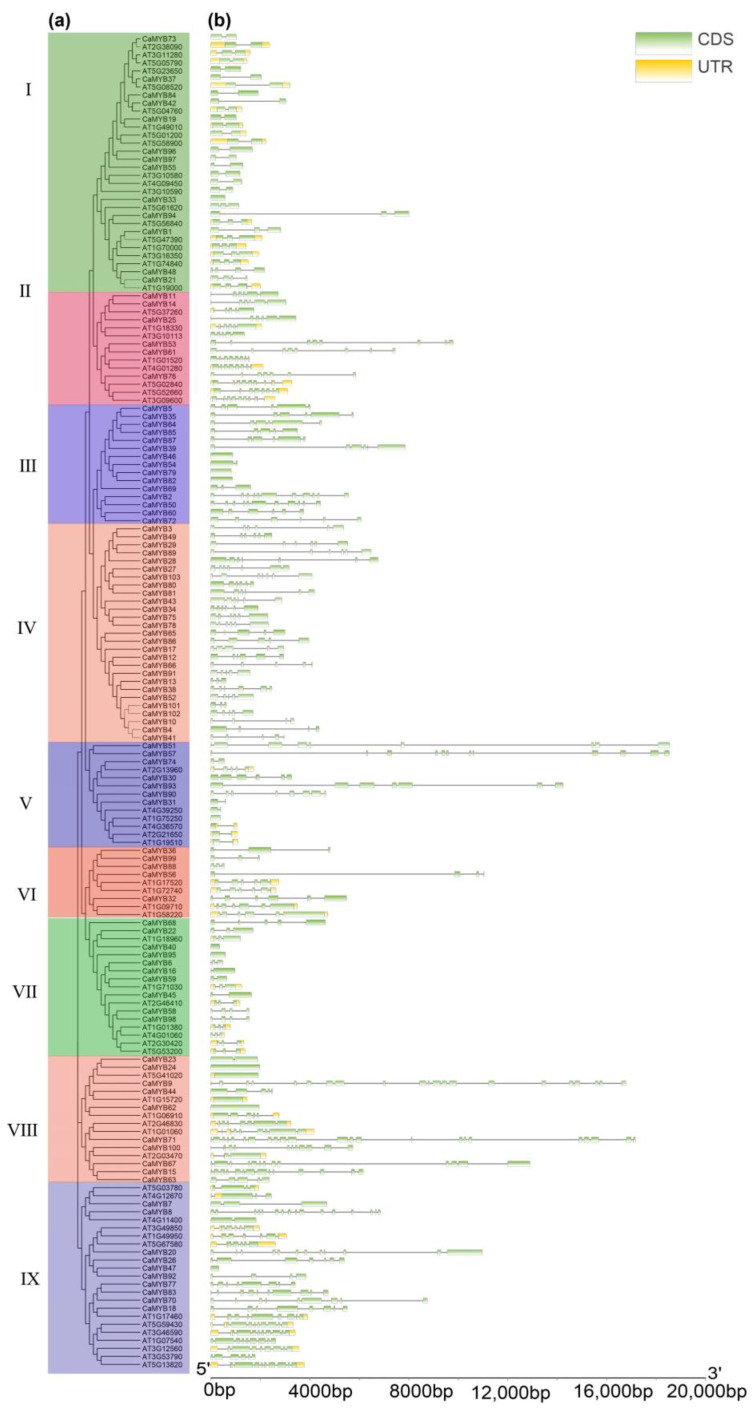
Phylogenetic relationship and gene structure of CaMYB-related and AtMYB-related families. (**a**) Unrooted phylogenetic tree of CaMYB-related and AtMYB-related proteins constructed using neighbor-joining method (NJ). (**b**) Gene structure. Green squares represent introns of genes, yellow squares represent uncoded UTR regions, and black lines represent exons.

A total of 103 *CaMYB*-related genes were divided into nine subfamilies according to the phylogenetic relationship and with reference to the classification of the *Arabidopsis* MYB-related family (Figure 3a). The gene structure analysis showed that the *CaMYB*-related genes have 0–22 introns, among which *CaMYB71* has the most introns (22) (Figure 3b). Ten conserved motifs (motifs 1–10) were identified in *CaMYB*-related members (Supplementary Figure S1). Each family member contained 1–6 motifs (Supplementary Figure S2). Members within the same subfamily had similar motifs. Except for individual members in subgroup VII, the remaining members contained motif 2, which is an important part of the conserved domain of the CaMYB-related family. A few subfamily members had unique motifs. For example, all genes in subfamily I contained motif 7, which was not found in other subfamily members. These genes may have unique functions.

### 2.5. Cis-Element Analysis of CaMYB-Related Family

**Figure 4:**
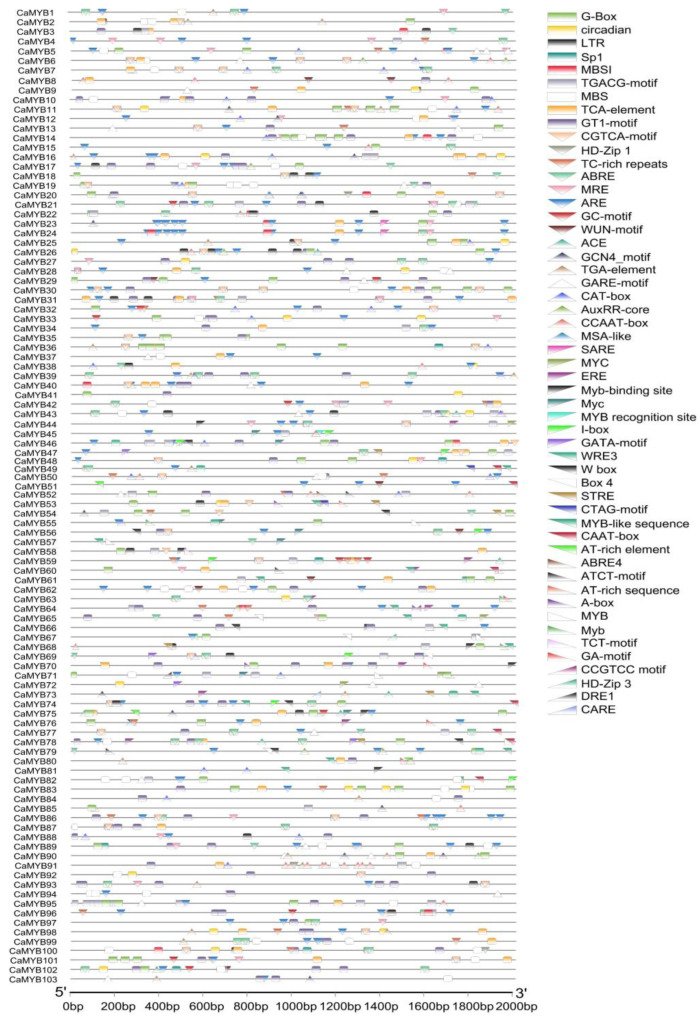
*cis*-Element analysis of CaMYB-related family. Different shapes and colors represent different elements.

The 2000 bp promoter sequences of the *CaMYB*-related genes were extracted, and the *cis*-regulatory elements (REs) in the promoters were predicted using the PLANTCARE online website (http://bioinformatics.psb.ugent.be/webtools/plantcare/html/, accessed on 5 May 2022). We identified 51 REs in the promoters of *CaMYB*-related genes. Among these, 20 REs are involved in light responses, such as G-box, CARE, and DRE1. Sixteen REs are involved in the regulation of abscisic acid (ABA), jasmonic acid (JA), and salicylic acid (SA) signaling pathways, including ACE, MYC, and ERE. The remaining REs are involved in biological processes such as stress responses, cell development, and metabolic regulation (Figure 4).

In parallel, we closely examined the promoters of the 103 *CaMYB*-related genes. Fifty-eight promoters contain elements involved in the regulation of flavonoid biosynthesis (TGACG), and 95 promoters contain palisade mesophyll cell differentiation-related elements (AREs). Elements involved in circadian rhythm control (LTR) in plants were identified in 25 promoters; 13 promoters contain WRE/STRE, a necessary element for anaerobic induction regulation, whereas 10 promoters contain hypoxia-specific induction element CGTCA motif. A total of 55, 60, and 23 promoters contain the CGTCA motif, GT1 motif, and TGA element, respectively, which are involved in the drought stress process (Supplementary Table S4). The above results indicate that the diversification of *CaMYB*-related family gene functions plays an important role in plant growth and development.

### 2.6. Expression Patterns of CaMYB-Related Genes in Various Tissues

**Figure 5:**
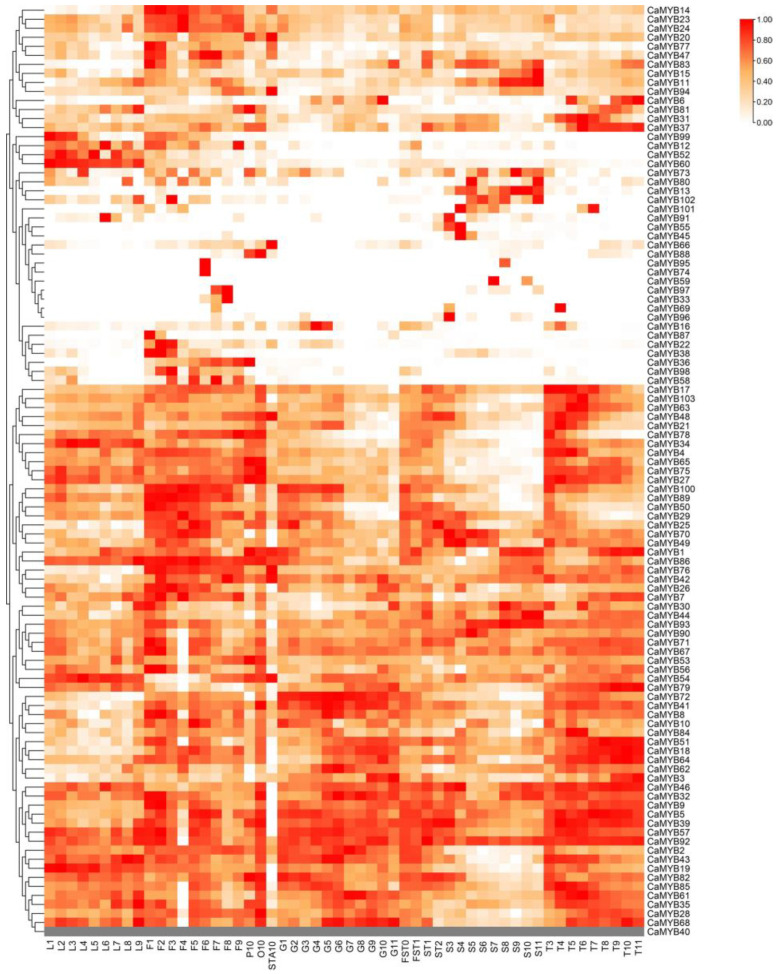
Expression patterns of *CaMYB*-related genes in different tissues and organs of pepper based on transcriptome data. L, F, O, G, S, and T represent the leaf, flower, ovary, pulp, seed, and placenta, respectively. ST denotes early seed and placenta, FST denotes early whole fruit, P10 denotes petal, and STA denotes stamen. Dark colors indicate high expression levels, and light colors indicate low expression levels.

We obtained transcriptome data of *CaMYB*-related genes in different tissues from previous studies and plotted the expression heatmap (Figure 5, Supplementary Table S5). Some *CaMYB*-related genes were expressed throughout all developmental stages and tissues, such as *CaMYB20/CaMYB27/CaMYB30/CaMYB63/CaMYB100*, while some displayed unique expression patterns. For example, *CaMYB52/CaMYB60/CaMYB99* were highly expressed in leaves, while *CaMYB14/CaMYB23/CaMYB24* were enriched throughout floral development, and *CaMYB22* was restricted to the early stage of floral development. Similarly, while *CaMYB13* was highly expressed throughout seed development, *CaMYB15* was restricted to late developmental stages. These unique expression patterns reflect their functions in different biological and developmental processes. In this study, we focused on *CaMYB37*, which is specifically and highly expressed in developing placenta. We speculated that *CaMYB37* may play an important role in regulating capsaicin biosynthesis.

### 2.7. Homology and Subcellular Localization of CaMYB37

**Figure 6:**
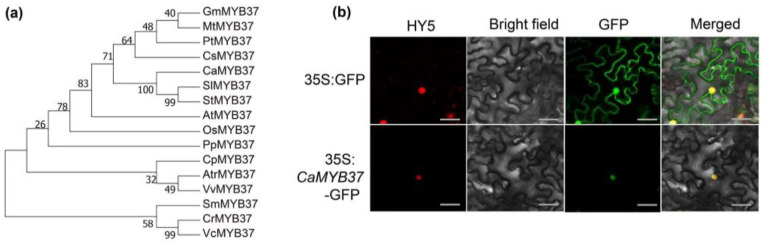
Phylogenetic analysis and subcellular localization of CaMYB37. (**a**) Phylogenetic analysis of amino-acid sequences of CaMYB37 in 16 plants. (**b**) Subcellular localization of the CaMYB37 protein in *N. benthamiana* leaves. Green fluorescent protein (GFP) signals indicated that CaMYB37 was localized in the nucleus. HY5 was used as a nuclear marker. Scale bar, 15 μm.

A phylogenetic tree was constructed by comparing the protein sequence of CaMYB37 with homologous protein sequences from other species (such as tomato and *Arabidopsis* 15 species). We found that CaMYB37 is closely related to SlMYB37 and StMYB37 (Figure 6a). However, there are few studies on the function of *MYB37* in Solanaceae. Subcellular localization analysis of CaMYB37 was conducted in *N. benthamiana* leaves. As shown in Figure 6b, 35S:CaMYB37-GFP colocalizes with HY5, which is used as a marker for nuclear proteins. Therefore, CaMYB37 is localized in the nucleus.

### 2.8. Silencing CaMYB37 Reduces the Expression of CBGs and Capsaicin Content

**Figure 7:**
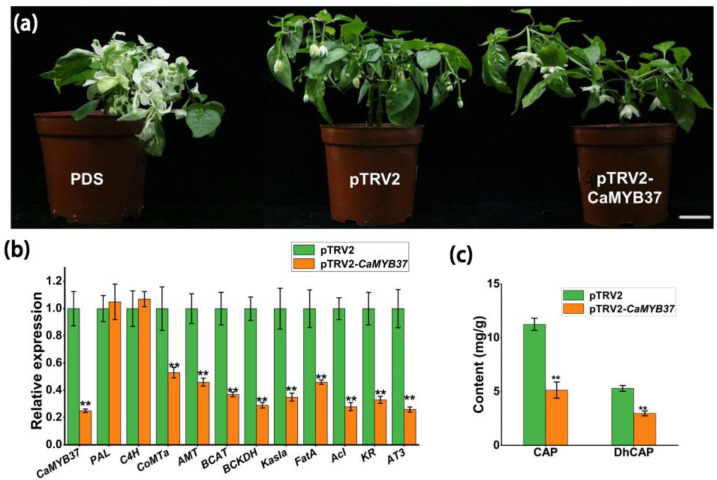
Expression level of *CBGs/CaMYB37* and contents of CaP/DhCaP in the control (empty vector) and *CaMYB37*-silenced fruit placenta. (**a**) Phenotypes of control, pTRV2-*PDS*, and pTRV2-*CaMYB37* plants. Scale bars, 10 cm. (**b**) Relative expression of *CaMYB37* and *CBGs* in the placenta at developmental stage 16 DPA in the *CaMYB37*-silenced fruit compared to levels in the control. The relative expression levels of the *CBGs* and *CaMYB37* in the control were set to 1, and those in the *CaMYB37*-silenced fruit placenta were measured relative to the control. (**c**) Contents of CaP and DhCaP in the control and *CaMYB37*-silenced fruit placenta. Error bars indicate means ± SEs (*n* = 3). ** means extremely significant difference (*p* < 0.01).

Gene silencing of *CaMYB37* in pepper cultivar ‘S8′ was performed using virus-induced gene silencing (VIGS). After about 1 month, the pepper plants injected with the *PDS* solution turned white, indicating that the VIGS test was successful (Figure 7a). The fruit placentas at 16 days after flowering were selected for qRT-PCR verification (Figure 7b). Compared with control plants, the expression levels of *CaMYB37* and capsaicin synthesis genes including *AMT*, *4CL*, and *AT3* were significantly decreased. At the same time, the contents of Cap and DhCap in fruit placentas at 45 days after flowering were determined. The contents of Cap and DhCap were significantly reduced in the placentas of pTRV2-*CaMYB37* plants compared with those in control pTRV2 plants (Figure 7c). Therefore, we can conclude that *CaMYB37* activates the expression of key *CBGs* and leads to increased capsaicin content in pepper.

### 2.9. CaMYB37 Binds to Capsaicin Synthesis Genes and Activates Their Transcription

**Figure 8:**
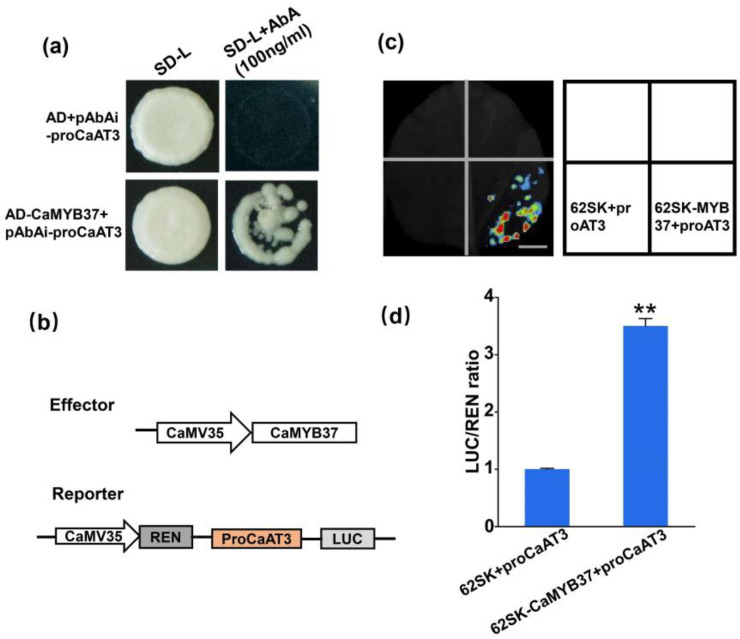
CaMYB37 binds to the promoter of *AT3* and promotes its transcription. (**a**) DNA-binding potential of CaMYB37 with the promoter of *AT3* in yeast. SD/−L, medium lacking leucine. (**b**) Vectors for dual luciferase experiments. (**c**) LUC images of tobacco leaves after transient infiltration. Scale bars, 1 cm. (**d**) Enzyme activity detections in dual luciferase assays. Error bars indicate means ± SEs (*n* = 6). ** means extremely significant difference (*p* < 0.01).

The *AT3* gene encodes capsaicin synthase, which catalyzes the synthesis of capsaicin from vanillylamine and 8-methyl-6-nonenoyl-CoA [27]. According to the PLANTCARE online tool, MYB, MBS, and other elements may be bound by the MYB proteins in the 2000 bp promoter sequence upstream of *AT3*. Therefore, a yeast one-hybrid experiment (Y1H) was performed to verify whether CaMYB37 binds to the *AT3* promoter. Yeast strain Y1HGOLD (pGADT7-CaMYB37) was co-transformed with pAbAi-*AT3*, and the transformants survived on selective medium lacking leucine and containing AbA, suggesting that CaMYB37 binds to the promoter of *AT3* (Figure 8a). To validate this observation, we performed a dual-luciferase assay (Figure 8c). The ratio of LUC to REN in tobacco leaves injected with 62SK-MYB37 and pro *AT3*-LUC increased 2.5-fold relative to the negative control (62SK and proAT3-LUC) (Figure 8d). The results indicate that CaMYB37 directly binds to the promoter of *AT3* and activates its expression in the pepper placenta.

## 3. Discussion

The MYB-related family has been reported to be involved in a wide range of biological processes such as circadian rhythm, hypocotyl elongation, meristem formation, and secondary metabolism [28,29,30,31,32]. The MYB-related family has been identified in *Arabidopsis* [15], tomato [33], rice [34], wheat [35], watermelon [36], eggplant [37], and other species. Previously, 235 *CaMYB* genes were identified in pepper [38]. Here, we used bioinformatics to identify members of the MYB-related family in pepper and analyze their evolutionary relationships, chromosomal locations, promoter elements, and expression patterns in various pepper tissues. A family member, *CaMYB37*, which is specifically expressed in pepper fruit placentas and flowers, was identified on the basis of transcriptome data. Gene silencing, yeast one-hybrid, and dual-luciferase reporter assays showed that *CaMYB37* regulates capsaicin biosynthesis by binding to the *AT3* promoter and activating its expression. In this study, the systematic analysis and functional verification of *MYB*-related family members can provide a foundation for the further exploration of *CaMYB*-related transcription factors in regulating pepper growth and development.

We identified 103 *CaMYB*-related members in the pepper genome. According to the taxonomy and phylogenetic relationship of *Arabidopsis* members, pepper *MYB*-related genes were divided into nine subgroups. Subfamilies III and IV consist only of pepper members. This may indicate the unique functions of these genes evolved in pepper [39]. In terms of gene structure, obvious differences were found in the number of exons among members of different *CaMYB*-related subfamily members, which may have led to functional differentiation during evolution. However, the number of exons within members of the same subfamily was not very different, indicating that the functions of these genes are conserved to a certain extent. The promoter sequences of the *CaMYB*-related genes contain various types of elements, such as light response, drought stress, and circadian rhythm regulation. Various types of elements have indicated that the *CaMYB*-related genes may play an important role in pepper growth, development, and stress response [40].

Homologous genes in the same branch of the evolutionary tree usually have certain similarities in plant functions. Many studies have been conducted on *Arabidopsis* MYB-related genes, allowing us to predict the function of the corresponding *CaMYB*-related homologous genes according to the known function of *Arabidopsis MYB*-related genes. *AtMYB*-related members of subfamily II are involved in the regulation of circadian rhythms in *Arabidopsis thaliana*, dormant germination of plant seeds, and flower bud development. For example, AT5G37260, AT1G18330, and AT3G10113 contain motifs 2, 1, and 5, respectively, and they are arranged in the same order. Previous studies have shown that *AT1G18330* (*EPR1*) encodes a nuclear-localized protein regulated by phytochromes A and B. Overexpression of *EPR1* increased far-red light-induced cotyledon opening and delayed flowering. *AT5G37260* (*RVE2*), *RVE7*, and *gibberellin 3-oxidase 2* (*GA3ox2*) act downstream of *FHY3*, and the transcriptional cascade composed of phyB-FHY3-RVE2/RVE7/SPT-GA3ox2 can transmit ambient light signals to regulate seed dormancy and germination [41]. *AT2G46410* (*CPC*), *AT1G01380* (*ETC1*), *AT2G30420* (*ETC2*), *AT4G01060* (*CPL3*), and *AT5G53200* (*TRY*) in subfamily VII mediate the differentiation of root hair cells, affecting the density of root hairs, development of trichomes in plants, and development of the waxy layer of the mustard epidermis [42]. The same subgroup of genes *CaMYB53*, *CaMYB61*, and *CaMYB76* may also be involved in the regulation of pepper root hair density and fruit epidermal cell development. *AT5G47390* (*MYBH*) and *AT1G70000* (*MYBD*) affect the accumulation of the secondary metabolite anthocyanin [43]. CaMYB1 of the same subfamily has similar gene domain structures; hence, *CaMYB1* may regulate anthocyanin synthesis in pepper.

Capsaicin is an important alkaloid that exists specifically in pepper, and the placenta is the main tissue producing capsaicin. Several studies have shown that *R2R3-MYB* family members regulate capsaicin synthesis in the placenta. For example, the R2R3-MYB-type transcription factor *MYB31* encoding Cap1 is specifically expressed in pepper placenta. However, no placental-specific expression has been found in other Solanaceae members such as tomato or potato, indicating that the tissue specificity of *MYB31* may be a consequence of capsaicin production unique to pepper placenta [18]. *MYB31* can directly upregulate the expression of CBGs in the placenta to increase capsaicin content. In another study, the transcriptome analysis showed that the expression of the *R2R3-MYB* transcription factor *CaMYB108* was higher in pepper fruits and flowers than in other tissues. Gene silencing (VIGS) and temporal overexpression assays of *CaMYB108* showed that this transcription factor was positively correlated with the content of capsaicin [44]. In the present study, through bioinformatic predictions, we identified *CaMYB37*, which was specifically expressed in the placenta. Our functional and molecular studies showed that *CaMYB37* promotes capsaicin biosynthesis by positively regulating the expression of several key capsaicin biosynthetic genes. Therefore, whether the identified seed-specific expression genes *CaMYB15* and *CaMYB13*, flower-specific expression genes *CaMYB23/CaMYB24*, and leaf-specific expression genes *CaMYB52/CaMYB60/CaMYB99* play an important role in the development of corresponding tissues deserves further study.

## 4. Materials and Methods

### 4.1. Identification of the CaMYB-Related Gene Family

Pepper and tomato gene and protein sequences were downloaded from the SGN website (https://solgenomics.net/, accessed on 5 May 2022). The *Arabidopsis* MYB-related protein sequences were downloaded from the TAIR website (https://www.Arabidopsis.org/, accessed on 1 May 2022). HMMER 3.0 [45] was used to screen tomato and pepper protein sequences containing the complete MYB domain (PF00249), with thresholds set at *E*-values of <1 × 10^−5^. Candidate sequences were further screened using the SMART website (http://smart.embl-heidelberg.de/, accessed on 1 May 2022) and the NCBI database (http://www.ncbi.nlm.nih.gov/, accessed on 1 May 2022), thereby retaining sequences containing the complete MYB domain. Candidate gene sequences were used for further analyses.

### 4.2. Phylogenetic Analysis and Classification of the CaMYB-Related Gene Family

The protein sequences of *Arabidopsis thaliana* and pepper MYB-related family members were subjected to multiple sequence alignment using ClustalW 2.0 [46]. A phylogenetic tree was constructed using the neighbor-joining (NJ) method in MEGA 7.0 with the following parameter settings: 1000 bootstrap values, Poisson model, and pairwise deletion [47]. The members of the CaMYB-related family were divided into different subfamilies based on AtMYB taxonomy and bootstrapping values in the phylogenetic tree.

### 4.3. Protein Properties, Conserved Motifs, and Gene Structures of CaMYB-Related Family

CaMYB-related proteins were uploaded to the ExPaSy website (http://web.expasy.org, accessed on 3 May 2022) to calculate the isoelectric point (PI) and molecular weight (MW). At the same time, MEME v5.1.1 (http://meme-suite.org/, accessed on 3 May 2022) was used to identify the conserved motifs of the CaMYB-related proteins. The optimal motif length was 100–200 residues, the number of motifs was 10, and the remaining parameters remained unchanged [48]. The gene structure and intron number of the pepper and tomato genes were determined with reference to the annotation files. Gene structure maps were drawn using TBtools v1.098765 [49].

### 4.4. Chromosomal Location and Gene Duplication Analysis of CaMYB-Related Family

MapGene2 (http://mg2c.iask.in/mg2c_v2.0/, accessed on 4 May 2022) was used to determine the location of the *CaMYB*-related genes on the chromosomes. MCScanX was used to identify *MYB*-related gene pairs in tomato, *Arabidopsis*, and pepper, as well as intraspecific gene pairs in pepper. The parameters were set as follows: match score of 50, match size of 5, gap score of −3, and *E*-value of 1 × 10^−10^. A collinearity graph was drawn using Circos software v0.69 (accessed on 5 May 2022) [50].

### 4.5. Analysis of cis-Elements in the Promoters of CaMYB-Related Genes

The 2000 bp upstream promoter sequences of the *CaMYB*-related genes were extracted using the SeqKit v0.13.0 software (accessed on 5 May 2022) [51]. After uploading the promoter sequences to PlantCARE [52] for online analysis, predicted promoter *cis*-elements were sorted and classified.

### 4.6. Expression Pattern Analysis of CaMYB-Related Genes

Previously reported transcriptome data were used to analyze the expression patterns of *CaMYB*-related genes in multiple organs and tissues of pepper [53]. The FPKM value (fragments per kilobase of transcript per million mapped reads) represents the expression level of each gene, which was converted to log_2_ (FPKM + 1). A heatmap was drawn after normalizing the data using R v3.6.1 (accessed on 7 May 2022).

### 4.7. Quantitative Real-Time PCR (qRT-PCR)

The fruit stage used in the experiment was 16 days after pollination [18]. Total RNA from ‘S8′ placenta was extracted using the TransZol kit (TransGen Biotech, Inc., Beijing, China). Each group of samples was repeated three times. Extracted RNA was reverse-transcribed using HiScriptIIQ RT SuperMix, and the product from the previous step was used in the qPCR (+gDNA wiper) vazyme kit (Vazyme, Piscataway, NJ, USA). qRT-PCR experiments were performed using a LightCycler^®^ 96 real-time PCR machine. The reaction volume was 20 µL, and the program was as follows: pre-denaturation at 95 °C for 30 s, 95 °C for 10 s, and 60 °C for 30 s, over 40 cycles, followed by 95 °C for 15 s, 60 °C for 60 s, and 95 °C for 15 s. Gene expression level was calculated using the 2^−ΔΔCt^ method [54]. qRT-PCR primers (*PAL/C4H/CoMTa/AMT/Kasla/BCAT/BCKDH/FatA/Acl/AT3/KR/Actin2*) were from the previous study [18]. The qRT-PCR primers of *CaMYB37* were designed by Primer5 software, and the specificity of primers was confirmed using Primer-BLAST (NCBI). The melting curve and standard curve were supplemented to check the amplification efficiency of qRT-PCR primers (*CaMYB37*) (Supplementary Table S6). The internal reference gene for gene expression levels was *Actin2* [18]. *Actin2* encodes an actin expressed during reproductive development. The gene primers used in this study are list in Supplementary Table S6.

### 4.8. Subcellular Localization

First, a subcellular localization expression vector was constructed. After double-enzyme digestion of the pSuper1300-eGFP plasmid, the obtained fragment was homologously recombined with the gene CDS sequence and then transformed into DH5α competent cells. A single colony was picked and shaken for sequencing, and the extracted plasmid was transferred into *Agrobacterium* competent GV3101. *Agrobacterium* liquid PCR test strips were expanded in LB medium containing kanamycin and rifampicin antibiotics. After 12 h, the OD600 was detected as 0.8–1. The sample was centrifuged at 3000 rpm for 8 min, resuspended in buffer (10 mM MES, 10 mM MgCl_2_, 100 mM AS), and incubated at 28 °C for 3 h in the dark. pSuper1300-CaMYB37-eGFP and the nuclear marker HY5-mCherry were transiently co-transformed into *Nicotiana benthamiana* leaves. The infected tobacco was cultured in an artificial climate chamber (day/night temperature 18/22 °C, light/dark 8/16 h). After 48 h, the fluorescence was detected using a laser confocal microscope and photographed (LSM800, Zeiss, Jena, Germany).

### 4.9. Extraction and Detection of Capsaicin and Dihydrocapsaicin

The capsaicin and dihydrocapsaicin contents in ‘S8′ placenta were extracted and detected using liquid chromatography tandem mass spectrometry (LC–MS/MS). The fruit stage used in the experiment was 45 days after pollination [18]. Cryopreserved pepper placenta samples were collected and ground into a powder with a grinder. About 0.1 g of the powder sample was weighed into a 2 mL centrifuge tube, before adding 1.0 mL of methanol extract; then, ultrasonic extraction was performed at 4 °C, and the process was repeated three times. The leaching solution was centrifuged at 10,000 rpm for 10 min, concentrated with nitrogen, and dissolved in 200 µL of methanol. All samples were purified by solid-phase extraction (SPE) using an Oasis HLB RP column (30 mg, 1 mL), washed with 1 mL of 100% MeOH and 1 mL of deionized water, and then eluted with 1 mL of methanol to obtain the analytical solution. The analytical solution was then blown dry with constant nitrogen at 40 °C and redissolved in 100 μL of methanol. The diluted samples were analyzed using UPLC–MS/MS. Using the external standard method, the peak areas of all the detected samples were substituted into the standard curve equation for calculation. The absolute contents of capsaicin and dihydrocapsaicin in the actual samples were obtained. All assays were performed in triplicate.

### 4.10. Virus-Induced Gene Silencing

First, a gene silencing expression vector was constructed. The coding sequence of the *CaMYB37* gene was uploaded to the SGN VIGS website (https://vigs.solgenomics.net/, accessed on 15 May 2022). A specific 300 bp sequence was selected for cloning. *PDS* (*Phytoene desaturase*) was used as an indicative control gene. We digested the pTRV2 vector and performed ligation transformation. Subsequent experiments were performed after the vector was verified to be correct. First, the bacteria were shaken before injection and resuspended when the OD value was approximately 1.0. The resuspended solution was allowed to stand for 4 h in the dark environment at 25 °C and then mixed. The pTRV1 bacterial solution and the bacterial solution containing pTRV2 or pTRV2-*CaMYB37* were mixed at a 1:1 ratio to prepare for inoculation. The pepper seedlings were subjected to drought treatment for 2 days when the cotyledons were fully expanded, and no true leaves were formed. A disposable syringe was used to inject the mixture into the backs of the *capsicum* cotyledons. The injected peppers were returned to an artificial climate chamber for cultivation.

### 4.11. Yeast One-Hybrid

The Y1HGold yeast strain was used to prepare competent yeast (the Y1HGold strain was purchased from the company). The *CaMYB37* coding sequence was ligated into the PGADT7 (AD) vector using homologous recombination. Specific primers were designed to amplify the promoter sequence of the capsaicin synthesis gene *AT3*. The *AT3* promoter sequence was ligated to the pAbAi vector, linearized, and transformed into competent yeast cells. The correctly sequenced pAbAi-*AT3* vector can screen for the minimum inhibitory concentration of Aureobasidin A (AbA) according to plaque growth. This screening concentration was considered suitable for subsequent one-to-one interaction experiments. The abovementioned Y1HGold (pBait-AbAi) strain was inoculated on SD/−Ura solid plates, and positive plaques with diameters of 2–3 mm were selected and propagated in YPDA liquid medium. The AD-CaMYB37 plasmid and AD empty plasmid were transformed into the Y1HGold strain and spread on SD/Leu plates. Positive yeast plaques were inoculated onto SD/−Leu+AbA plates. After 3–5 days, plaque growth was observed.

### 4.12. Dual-Luciferase Reporter Gene Assay

Two types of vectors were constructed for the Dual-Luc reporter gene detection experiments. The cloned *AT3* promoter sequence was ligated into a pGreenIIo800 vector. The coding sequence (CDS) region of cloned *CaMYB37* was ligated into the pGreenII62-sk vector. The two vectors were transformed, and the tested bacterial liquid was expanded and cultured in LB medium containing kanamycin and rifampicin. The samples were cultured until the OD600 value reached 1. After centrifugation for 5 min at 5000 rpm, the bacteria were collected. The cells were resuspended in an MES solution (10 mM MES, 10 mM MgCl_2_, and 100 mM AS). The suspension was then mixed in the dark for 4 h (pGreenII0800:pGreenII62-sk = 1:9). A 1 mL syringe was used to inject the back of the leaves (tobacco seedlings were approximately 1 month old). The injection dose of each leaf in the control and experimental groups remained the same (1 mL). After injection, the tobacco plants were treated in the dark for 48 h. Fluorescence was observed using an in vivo imager. The leaves injected with the mixed solution were sampled using a hole punch, quick-frozen in liquid nitrogen, and ground. Enzyme activity was detected using a dual-luciferase reporter assay system kit. The primer pairs used for the yeast one-hybrid and dual-luciferase reporter assays are listed in Supplementary Table S1.

### 4.13. Statistical Analysis

Data were analyzed using GraphPad Prism software (version 7; San Diego, CA, USA) and the Student’s *t*-test for significance in all assays. Error bars indicate means ± SEs. “*” means significant difference (*p* < 0.05). “**” means extremely significant difference (*p* < 0.01).

## 5. Conclusions

In conclusion, we systematically identified and analyzed *CaMYB*-related family members. Through bioinformatic prediction, we identified placental-specific *CaMYB37*. We provided functional and molecular evidence that CaMYB37 regulates capsaicin biosynthesis by binding to the *AT3* promoter and activating its expression. The results provide reference for further study on the role of *CaMYB*-related genes in pepper growth and development.

## Data Availability

The data presented in this study are available in insert article or supplementary material here.

## Supplementary Materials

The following supporting information can be downloaded at: https://www.mdpi.com/article/10.3390/ijms231911667/s1.
