# Supplementary material for: Genome-Wide Analysis of the MYB-Related Transcription Factor Family in Pepper and Functional Studies of CaMYB37 Involvement in Capsaicin Biosynthesis
Source: Int J Mol Sci. 2022 Oct 1;23(19):11667. doi: 10.3390/ijms231911667 (PMC9569548; doi:10.3390/ijms231911667)
Supplement: Supplementary file 1 [file ijms-23-11667-s001.zip › Supplementary Materials captions edited.pdf]

Supplementary Figure S1 Sequence logos of conserved motifs of *CaMYB*-related genes.
Supplementary Figure S2 Phylogenetic relationship and motifs of CaMYB-related and AtMYB-related families. (a) Unrooted phylogenetic tree of CaMYB-related and AtMYB-related proteins constructed using neighbor-joining method (NJ). (b) Conserved motifs. Different colored squares represent different types of motifs.
Supplementary Table S1 *MYB*-Related genes in pepper, tomato, and *Arabidopsis*.
Supplementary Table S2 Basic information of the *MYB*-Related transcription factor family.
Supplementary Table S3 Syntenic *MYB*-Related gene pairs among tomato, pepper and *Arabidopsis*.
Supplementary Table S4 *cis*-Elements Analysis of the *MYB*-Related genes.
Supplementary Table S5 The FPKM values of *MYB*-Related genes in different tissues and organs.
(L, F, O, G, S, and T represent the leaf, flower, ovary, pulp, seed, and placenta, respectively. ST denotes early seed and placenta, FST denotes early whole fruit, P10 denotes petal, and STA denotes stamen. 1-10 in L1-L10 represent 2, 5, 10, 15, 20, 25, 30, 40, 50, and 60 days after the emergence of new leaves; 0 (FST) and 1 (FST) represent 3 and 7 days after pollination days; 1-11 (G, S, T) represent 10, 15, 20, 25, 30, 35, 40, 45, 50, 55, and 60 days after pollination.)
Supplementary Table S6 Primer sequences used in this study.
qPCR primers (*PAL*/*C4H*/*CoMTa*/*AMT*/*Kasla*/*BCAT*/*BCKDH*/*FatA*/*Acl*/*AT3*/*KR*/*Actin2*) were from the previous study [18].
