# Supplementary figures and images for: Genome-Wide Analysis of the MYB-Related Transcription Factor Family in Pepper and Functional Studies of CaMYB37 Involvement in Capsaicin Biosynthesis
Source: Int J Mol Sci. 2022 Oct 1;23(19):11667. doi: 10.3390/ijms231911667 (PMC9569548; doi:10.3390/ijms231911667)

motif1

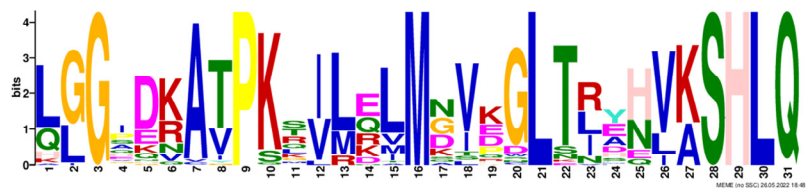

motif2

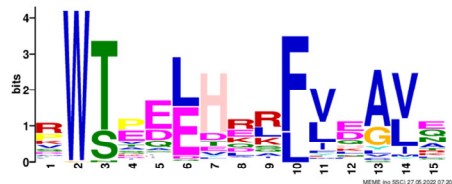

motif3

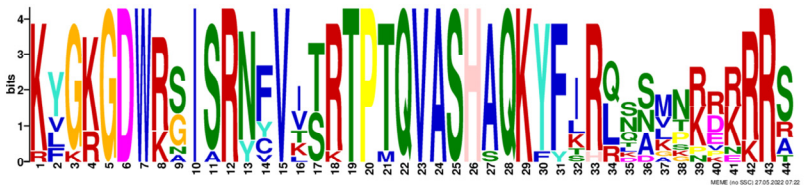

motif4

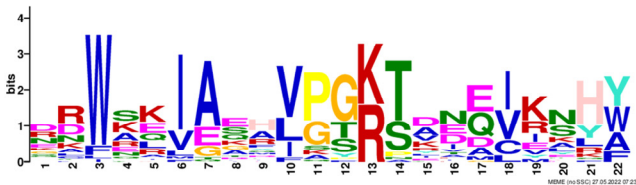

motif5

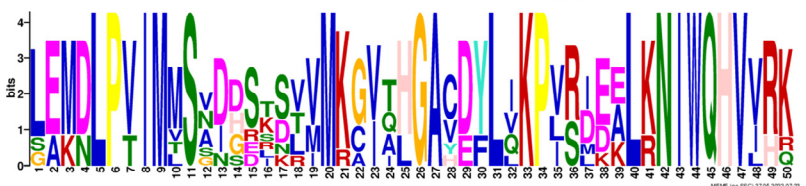

motif6

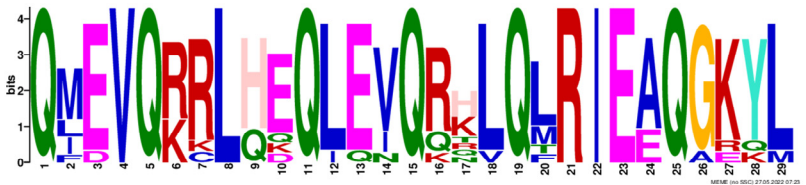

motif7

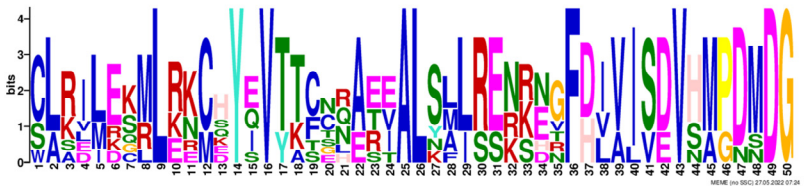

motif8

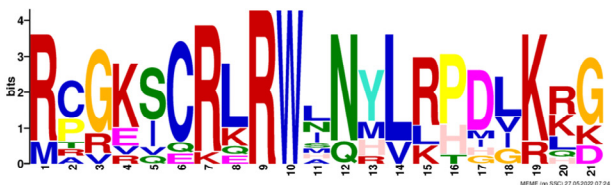

motif9

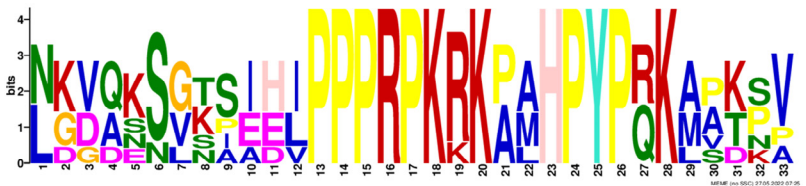

motif10

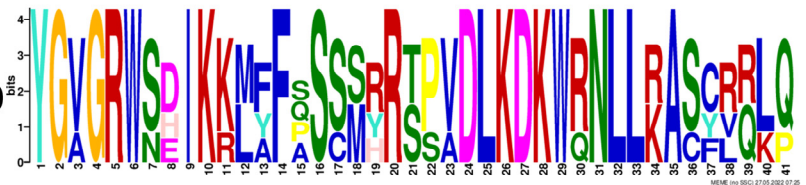

Supplement: Supplementary file 1 [file ijms-23-11667-s001.zip › SupplementaryFigure S1.pdf]

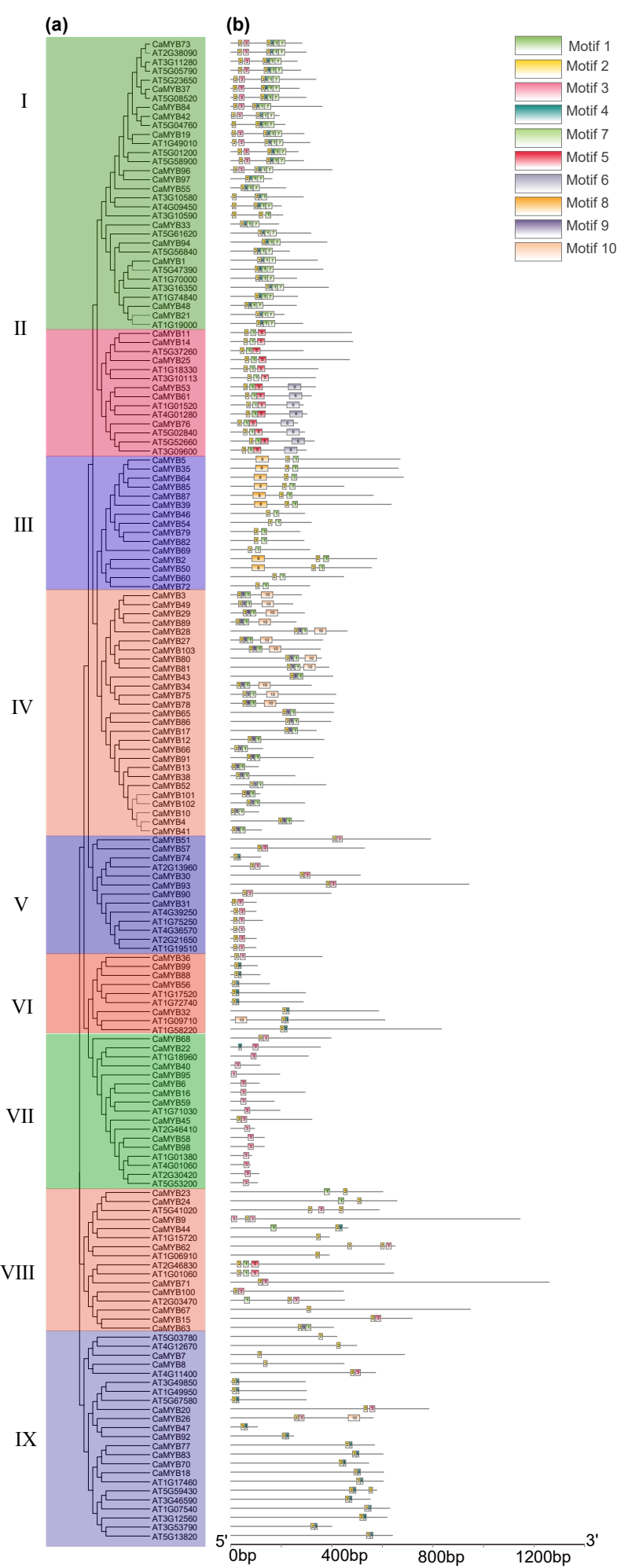

Supplement: Supplementary file 1 [file ijms-23-11667-s001.zip › SupplementaryFigure S2.pdf]
